# In Search for the Meaning of Illness: Content of Narrative Discourse Is Related to Cognitive Deficits in Stroke Patients

**DOI:** 10.3389/fpsyg.2020.548802

**Published:** 2021-01-18

**Authors:** Anna R. Egbert, Agnieszka Pluta, Joanna Powęska, Emilia Łojek

**Affiliations:** ^1^Faculty of Medicine, University of British Columbia, Vancouver, BC, Canada; ^2^Faculty of Psychology, University of Warsaw, Warsaw, Poland; ^3^Bioimaging Research Center, World Hearing Center, Institute of Physiology and Pathology of Hearing, Warsaw, Poland

**Keywords:** cognitive function, neuropsychological assessment, narrative medicine, stroke, discourse

## Abstract

Stroke survivors undergo a thorough cognitive diagnosis that often involves administration of multiple standardized tests. However, patient’s narrative discourse can provide clinicians with additional knowledge on patient’s subjective experience of illness, attitude toward current situation, and motivation for treatment. We evaluated the methods of analyzing thematic content and story types in relationship to cognitive impairment in stroke survivors with no aphasia (including 9 left hemisphere damage – LHD patients, and 16 right hemisphere damage – RHD patients). Cognitive impairment was evaluated in comparison to a group of 25 patients with orthopaedic injury not involving the brain. Our findings primarily show that higher elaboration on own cognitive problems, physical ailments or coping strategies in LHD patients and cognitive problems, emotional issues and circumstances of illness onset in RHD patients is related to deficits in executive functions and retrieval of information from memory. Furthermore, RHD patients who use more chaos story type show lower executive functioning. However, these results did not survive the significance threshold of *p* < 0.05 after Bonferroni adjustment for multiple comparisons. In conclusion, this study provides preliminary evidence that stroke survivor’s narrative can constitute an additional source of clinically-relevant information regarding patient’s experience of illness and attitude toward recovery. This knowledge can aid clinicians and nurses in everyday interactions with the patients and support individualized strategy to treatment. Still, the current results need be confirmed with future studies in a larger cohort of stroke patients.

## Introduction

Conversation between the clinician and the patient should be the entry point for neurorehabilitation (Christensen et al., 1989). A clinical interview can provide information regarding patient’s self-awareness, self-evaluation, and subjective complaints (Prigatano, 1999). Tools such as the Patient’s Assessment of Own Functioning Inventory (PAOFI) can aid in exploration of the patient’s subjective rating of preserved and impaired functional areas (Chelune and Lehman, 1986). However, eliciting patient’s narrative discourse can additionally allow clinician to gain access to the patient’s subjective realm (van der Riet et al., 2011a), motivations for treatment, and meaning of illness that are influenced by the patient’s social and cultural background (Hyden, 1997).

Narrations refer to discourse that has a personal meaning and is organized around sequential events (Hjelmblink and Holmstrom, 2006). In the diagnostic setting, narrations can be elicited in stroke survivors with the use of an open-ended interview (McKevitt, 2000; Pluta et al., 2015). There are existing approaches to investigate narrative ability, structural aspects (Marini, 2012), and thematic content of narrations in stroke survivors (Pluta et al., 2015). The aim of assessing narrative ability and structural aspects is to display intricate micro- and macrolinguistic impairments as well as preserved aspects of language function in stroke survivors (Marini, 2012). This method helps to build a clinical portrait of the abilities of the stroke survivor to organize information and communicate it in a coherent and informative manner (Marini, 2012). This aim is radically different from analyzing narrative discourse in order to uncover a patient’s subjective perspective, experiences, attitudes, or beliefs. The “thematic framework of illness narratives” method analyzes the content of stroke patients’ discourse (Pluta et al., 2015). In this method, every narration is split into utterances, which are then categorized by themes (Pluta et al., 2015). This type of analysis of patient discourse can be a useful tool for clinicians to understand patient’s state of knowledge related to illness and socio-psychological state and may help in planning the course of rehabilitation (Pluta et al., 2015).

However, analyses of patient illness narrations can reach beyond the structural or thematic levels and disclose patients’ approach to reconstructing (or restructuring) themselves in the context of illness (Frank, 1995). Clinicians can achieve this level of understanding by using Frank’s story typology (1995). This method distinguishes three story types, i.e., restitution, quest, and chaos. In the restitution story type, the focus is on getting back to health. It is culturally preferred and the most commonly used story type, especially in the recently ill (Frank, 1995). In the quest story type, patients express that they have accepted their illness with its consequences and are ready to gain something new out of their current state. In the chaos story type, patients show lost hope for their life ever getting better. These narratives may even seem threatening or anxiety-provoking (Frank, 1995). Previous research shows that patients may use the chaos narrative as a powerful tool to express suffering and related experiences (Hyden, 1997).

In stroke and breast cancer survivors, Frank’s story typology (1995) is shown to be a useful tool in identifying the dominant narrative genres (France et al., 2013). Existing literature describes that the dominant narrative genre is greatly influenced by the severity of stroke and actual or anticipated recovery (France et al., 2013). Patients with mild-to-moderate disability mostly produce restitution and quest memoir narratives (France et al., 2013). Patients who are the most disabled and show little improvement post-stroke tend to produce more chaos narratives (France et al., 2013). Nonetheless, the relationship between the use of a particular story type and the severity of global cognitive impairment as well as deficits in particular cognitive domains remains unclear.

Cognitive deficits in stroke survivors vary and depend on multiple factors, such as age, previous history of stroke, pre-stroke functional level, and location or size of brain damage (Nys et al., 2005; Su et al., 2015). A wide range of cognitive functions may be affected: attention (Loetscher and Lincoln, 2013), executive functions (Motta et al., 2014), language (Watila and Balarabe, 2015), learning (Andrews et al., 2014), memory (Elliott and Parente, 2014), perception (Duclos et al., 2014), or processing speed (Su et al., 2015). Right-hemisphere stroke is primarily linked to visuospatial neglect (Stone et al., 1993). Stroke in the left (or language-dominant) hemisphere frequently results in disorders of production and/or comprehension of language (Watila and Balarabe, 2015). Language production and comprehension deficits can also be secondary to the impairments in the cognitive functions that support them, including a wide range of attention abilities and executive attention processes (for review, see Barker et al., 2020). However, even stroke survivors with obvious symptoms of aphasia may show narrative achievement and preserved pragmatic abilities (Ulatowska et al., 2010; Pluta et al., 2015). Furthermore, many stroke survivors do not show aphasia and are able to produce cohesive narrative discourse (Ellis et al., 2005; Marini, 2012). In non-aphasic patients, subtle expressive language disruptions diminish over time (Ellis et al., 2005). Yet, the scientific literature on the relationship between narrative discourse and cognitive deficits other than production of language, especially in stroke patients with no aphasia symptoms, is unfulfilling.

This study aimed at evaluating the relationship between thematic content of narrative discourse, story type, and cognitive functions in stroke survivors with no aphasia. We propose that the thematic framework of illness narratives (Pluta et al., 2015) and story typology (Frank, 1995) can be useful tools for the purpose of analyzing narrations in stroke survivors with left- and right-brain hemisphere damage. We hypothesized that the extent to which patients elaborate on particular topics and use particular story types (i.e., restitution, quest vs. chaos) correspond with the severity of their deficits in particular cognitive domains. In particular, we expected that patients who express higher preoccupation with their own state of health and lost hope for getting better will also show more compromised cognitive functions. The Ethics Committee of the Faculty of Psychology, University of Warsaw, approved the study.

## Materials and Methods

### Participants

Twenty-five stroke survivors were recruited from the inpatient rehabilitation program at the Neurological Rehabilitation Centre in Konstancin-Jeziorna near Warsaw, Poland. Eligible participants suffered a single ischemic stroke in the left or right brain hemisphere, which was confirmed with neurological assessment (CT/MRI). Retrospective information on the symptoms of aphasia in the acute phase after stroke was obtained from patients’ medical history. At the time of recruitment for the study, aphasia symptoms were assessed with the Aphasia Severity Rating Scale (ASRS), the Boston Diagnostic Aphasia Examination (Goodglass and Kaplan, 1983), by a trained clinical neuropsychologist. Only patients who received an ASRS score ≥ 4 (meaning no significant disturbances in the production or comprehension of speech) were directed to the research personnel for possible participation in the current study. Inclusion criteria for the study were adult, single stroke, no observable aphasia symptoms (i.e., ASRS score ≥ 4), sufficient cognitive status to independently participate in the study (i.e., capable of giving informed consent), and able to operate at least one hand. Exclusion criteria were major depression or previous history of traumatic brain injury or neurological disorder.

The non-stroke comparator group was included in the study design in order to contrast the outcomes of standardized cognitive tests in stroke patients with individuals with similar life circumstances. Therefore, the inclusion criteria for the comparator participants were hospitalization due to orthopedic non-head injury, no history of stroke, and no neurological illnesses or depression. The comparator group was recruited from the Gruca’s Independent Public Orthopedic and Trauma Teaching Hospital, Poland. All participants gave informed consent.

### Interview

The experimental method of an interview was employed to elicit patients’ discourse. Participants were asked four open-ended questions in the following fixed order: 1. Can you tell me about your illness? 2. How has your life changed as a result of your illness? 3. Do you see any difficulties in yourself as a result of your illness? 4. Has anything got better since the onset of your illness? The researcher listened attentively without making any comments or interruptions to the patient’s discourse. The next question followed once the patient finished answering the previous one, i.e., explicitly stated they were finished, asked for the next question, or suggested it via non-verbal behavior. Interviews were registered on a voice recorder with participants’ word-of-mouth permission.

Questions #1 and #2 are evidence-based interview questions used in line with the previously described and implemented methodology of the thematic framework of illness narratives (Pluta et al., 2015). Questions #3 and #4 were developed for the purpose of the current study. In detail, the motivation behind adding questions #3 and #4 was to provide an opportunity for participants to talk about both the negative (question #3) and the positive sides (question #4) of their illness. It was important to elicit this additional discourse in order to provide bases for the analyses of chaos, quest, and restitution story types. Importantly, we decided to ask the questions in a fixed order: first question, enter the conversation with a general question about the illness; second question, focus the conversation on the changes caused by the illness; third question, elicit a narrative on the possible negative results of the illness; fourth question, refocus the conversation and elicit a narrative on the possible positive results of the illness. We asked the question about positive side effects of the illness as the last question of the interview with the purpose of reorienting the patient toward positive thoughts before ending the session. Additionally, clinicians, nurses, and other hospital staff were advised to monitor and alert the neuropsychologist and study personnel in case participants showed any symptoms of emotional burden, such as depressive symptoms, following the interview.

### Narrative Discourse Analysis: Thematic Content

Thematic content of narratives was investigated with the proportion of *utterances* on revealed *topics* using the methodology previously published by Pluta et al. (2015). *Utterance* is defined as a vocal expression containing one theme. For example, two succeeding utterances (separated with a full stop) were as follows: “It was a stroke…ischemic one. However, medical examination didn’t reveal any particular cause of it” (RHD, female, 59 years old). *Topics* were distinguished as particular issues disclosed by at least one third of participants. Issues that were reported by less than one third of participants or that were irrelevant to the illness were categorized as “other.” The list of topics identified in previous research is described elsewhere (Pluta et al., 2015). That list was suggested to the neuropsychologists who were rating the narratives as a guideline for potential topics based on previous literature (Pluta et al., 2015). However, the raters were instructed that, if an utterance did not match any of the topics from the list, they should categorize it as a new different topic. The outcome score for the thematic content was the proportion of utterances produced on particular topics that were identified in the current study sample.

### Narrative Discourse Analysis: Story Types

Narratives were analyzed according to Frank’s (1995) story typology in order to investigate patients’ attitude toward their own illness. Utterances that were identified with the thematic content analyses were categorized into either restitution, quest, or chaos story types in line with the description of each story type provided by Frank (1995) as follows. In the restitution story type, utterances concentrate on the patient getting well and taking restitution as a remedy for suffering from illness. The genesis of illness is not of concern. Instead, utterances focus on the moment just before the illness onset as well as the previous predictability of the body. Narrations express that it is the body but not the self that is ill. Utterances describe tests and interpretations, treatment options, possible outcomes, and doctors’ competence. Patients view themselves as being exempted from normal responsibilities and rather obliged to submit themselves to the authority of a recognized professional and comply with their indications in order to get well. Thus, restitution narrations are primarily about health.

In the quest story type, utterances express acceptance of the illness and its consequences. Patients’ utterances focus on alternative use of their illness, a new purpose, or a new quest to find how they can prosper given their new circumstances. Utterances show that patients consider themselves as “marked/reborn” or that they discover “who they always have been.” The quest stories resemble three stages of a journey described by Campbell (2004): (i) *departure* (early body signs of something not being right, often refused, denied), (ii) *initiation* (symptoms become too obvious to be mistaken, followed by a “road of trials” to obtain the diagnosis, atonement, final apotheosis, or gaining important self-knowledge), and (iii) *return* (being “marked” by the illness). There are also three facets of quest story, i.e., *memoir* (illness incorporated into other life events), *manifesto* (illness as a social issue, telling the truth of suffering is an obligation to the society), and *automythology* (patient is “reborn” after surviving illness; therefore, the change is underlined).

In the chaos story type, utterances are an “emotional talk” about the current situation without mentioning the past or future. The syntactic structure lacks narrative order or causality. Instead it involves frequent use of the phrase “and then” and silences. Utterances express the belief that life is never going to get better. They are threatening, anxiety provoking, and concerned with emotional battering. Patients focus on being incapable of taking control over their own life or illness and give examples of failure to restore contingency. The self is presented as dissociated from the body, and the body can no longer be controlled. Utterances also include lack of support, comfort, or recognition from other people.

Importantly, as described by Frank (1995), each participant’s narrative can contain all three story types. This means that, as the patient produces discourse, the patient switches between the three story types. Consequently, some utterances are on the verge of two story types and can contain characteristics of both of those story types. In cases in which no clear distinction could be made as characteristics of both story types were present, the raters were instructed to categorize such utterances as both story types.

The outcome score for story type analysis is the percentage of every story type in each participant’s narrative. We chose the story type outcome score to be the percentage of words that were produced using particular story type because the utterances vary in length, which can further reflect the extent to which participants elaborate on their illness narrative using a particular story type. Our decision was guided by increasing the sensitivity of the measure of story type. The following formula was applied to calculate the outcome score for each participant: (1) calculate the number of words produced using the restitution, quest, and chaos story types; (2) divide each of the obtained numbers in the step 1 by the total number of produced words; and (3) then multiply each of the numbers obtained in step 2 by 100.

However, the total percentage (i.e., sum of percentage of the three story types) may exceed 100 in some participants. That is possible as some utterances are on the verge of two story types when the patient switches between them in the narrative discourse. Those utterances can be assigned to either of those story types and, thus, are categorized as both. Further considerations on the possible adverse consequences of categorizing the same utterance as two separate story types are discussed in the Limitations section of the current paper.

### Inter-Rater Reliability Assessment

In order to assess inter-rater reliability for categorizing utterances into topics and story types, two raters independently analyzed 25 scripts. One rater was the neuropsychologist who administered the interview and the standardized neuropsychological tests in the current study. The second rater was a neuropsychologist who was not involved in the data collection and did not have contact with any of the participants. First, the two raters independently categorized each utterance into the topics as previously described (Pluta et al., 2015). Next, raters independently classified the utterances as restitution, quest, or chaos story types according to the characteristics previously described by Frank (1995). Finally, the independent ratings of topics and story types were entered into the Kendall *W* coefficient of concordance in order to examine the inter-rater reliability.

### Cognitive Assessment

An extensive battery of standardized neuropsychological tests was administered as follows. The Polish version of the Wechsler Adult Intelligence Scale-Revised – WAIS-R(PL; Brzeziński et al., 2004) was administered with the total score indicating general intellectual abilities and subtests assessing memory, thinking, visuo-spatial functions, activity rate, and attention. The battery was administered and evaluated due to standardized procedures with the exception of the Digit-Symbol Coding Test in patients showing unilateral inattention for whom post-omission patterns were also scored. The Polish version of the Right Hemisphere Language Battery (RHLB-PL; Bryan, 1994; Łojek, 2007) was used as a measure of abstract thinking beyond the information given in the exercise, actualizing their own knowledge and integrating data, efficiency of converting visuo-spatial data, non-verbal abilities engaged in analyzing language and non-verbal data, processing and analyzing verbal data completely included in the exercise, executive functions (monitoring abilities, controlling own behavior), understanding the emotional aspect of auditory data, and adequacy of verbal and non-verbal communication in a social setting. The Polish version of the California Verbal Learning Test (CVLT; Łojek and Stańczak, 2010) was used to assess verbal memory and learning functions. The Wisconsin Card Sorting Test (WCST; Jaworowska, 2002) was administered as a measure of executive functions. The Trail-Making Test (TMT; Reitan and Wolfson, 1985; Reitan and Wolfson, 2004) assessed activity rate (TMT-A) and executive functions (TMT-B). The Ruff Figural Fluency Test (RFFT; Łojek and Stańczak, 2004) measured non-verbal fluency. The Star version of the Test of Attention and Perception (*pl*. wersja gwiazd Testu Uwagi i Spostrzegawczości – TUS-gw; Ciechanowicz and Stańczak, 2006) was used to assess visual inattention. It is a 3-min task of crossing out two indicated star-shaped symbols placed among distracting stimuli that are graphically similar. The TUS-gw consists of 54 rows of symbols, each containing between 4 and 8 hits out of 18 stimuli. Finally, we used the Bells Test (Gauthier et al., 1989) to assess visual neglect by analyzing the unilaterality of spatial distribution of omissions.

For the current study, we retained particular indices from the cognitive tests to assess cognitive domains as follows. To examine abstract thinking and non-verbal abilities engaged in analyzing language and non-verbal data, we retained WAIS-R-PL Similarities outcome scores and the RHLB-PL Picture Metaphors Test and Inference Test. For attention and psychomotor speed, we retained TMT Trial A (i.e., time of completion). For executive functions, we retained the following WCST indices: number of correct responses, percentage of errors, percentage of perseveration errors, and percentage of conceptual answers. For language functions and understanding the emotional aspect of auditory data, we retained WAIS-R-PL outcome scores for comprehension as well as the RHLB-PL Lexical-Semantic Test and Emotional Prosody Test. For memory and verbal learning, we retained outcome scores for WAIS-R-PL Digit Span and the following CVLT indices: A1-5, free recall short delay, free recall long delay, and recognition. For visuo-spatial functions, we retained WAIS-R-PL outcome score for Visual Puzzles.

### Statistical Analysis

Narrative analysis commenced with the inter-rater reliability test, i.e., the Kendall *W* coefficient of concordance. Limitations to the inter-rater reliability are reported in the Limitations section of this article.

Because of the small sample size, variables for demographics, illness characteristics and raw cognitive scores were evaluated as to whether they met the assumptions for running parametric tests. Because the variables did not meet the assumptions for running parametric tests, further between-group comparisons and correlation statistics were performed using the non-parametric tests. Between-group differences in the number of utterances on the narratives’ main topics and the percentage of story types in the narratives were analyzed with the Mann–Whitney *U* test. We employed Spearman’s *Rho* to identify significant thematic content and story type correlates of cognitive function (i.e., raw scores on cognitive tests). Because of the high number of pairwise comparisons, we calculated Bonferroni-adjusted significance. Therefore, throughout the manuscript, we report whether the interpretation of particular statistical results is based on Bonferroni-adjusted or uncorrected *p*-values. All statistical computations were performed using SPSS version Statistics 19. Limitations due to interpreting uncorrected *p*-values are discussed in the Limitations section of this article.

## Results

### Sample Characteristics

Table 1 summarizes the demographic and clinical characteristics of the sample. Twenty-five individuals after stroke participated in the study. Out of those, 9 participants had left hemisphere damage (LHD, 75% male; age in years median = 57.00) and 16 had right hemisphere damage (RHD, 50% male; age in years median = 68.50). In addition, 25 orthopedic injury comparators (OIC, 52% male; age in years median = 63.00) participated in the study.

**TABLE 1:** Demographic and clinical characteristics of the sample.

	Brain hemisphere damage					
	Left (*n* = 9) Mdn	Right (*n* = 16) Mdn	OIC (*n* = 25) Mdn	LHD – OIC *U* (Bonferroni corrected *p*-value)	RHD – OIC *U* (Bonferroni corrected *p*-value)	LHD – RHD *U* (Bonferroni corrected *p*-value)
Age, Mdn	57.00	68.50	63.00	84.50 (0.822)	150.50 (0.555)	37.00 (0.141)
Male: female	6: 3	8: 8	13: 12	–	–	–
Education (years), Mdn	13.00	13.00	13.00	103.00 (1.000)	184.00 (1.000)	61.00 (1.000)
Right: left handedness	9: 0	16: 0	24: 1	–	–	–
Time since illness onset (years), Mdn	0.50	1.00	0.50	36.00 (0.852)	17.00 (0.024)	46.50 (0.411)
Rehabilitation period (months), Mdn	1.00	1.50	1.00	47.00 (1.000)	77.00 (1.000)	59.50 (1.000)
IB-ADL, Mdn	18.00	17.00	–	–	–	58.00 (1.000)
ASRS, Mdn	5.00	6.00	–	–	–	24.00 (<0.001)
Hemiparesis/hemiplegia of the dominant hand	4	0	–	–	–	
Neuroimaging results: - Hypotensive areas in FL, TL, PL of LH - Hypotensive areas in FL, PL of LH - Hypertensive areas in FL, TL, PL of LH - Hypertensive areas in FL, PL, insula of LH - Hypotensive areas in FL of RH - Hypotensive areas in FL, TL, PL of RH - Hypotensive areas in TL of RH - Hypotensive areas in RH, generalized cortico-subcortical atrophy, expansion of intracranial fluid spaces - Hypertensive areas in FL, TL, PL of RH - Hypertensive areas in FL of RH - Multifocal vasogenic brain damage - Vasogenic leukoaraiosis in FL, PL - Not assessed	1 2 1 1 – – – – – – 1 – 3	– – – – 2 1 2 1 1 1 2 1 5	– – – – – – – – – – – – 25			

*Note. LHD, left-hemisphere damage patients; RHD, right-hemisphere damage patients; OIC, orthopedic injury comparators; Mdn, median; LH, left hemisphere; RH, right hemisphere; FL, frontal lobe; TL, temporal lobe; PL, parietal lobe; ASRS, Aphasia Severity Rating Scale; and IB-ADL, Barthel Index of Activities of Daily Living. The presented p-values are Bonferroni-adjusted for multiple comparisons.*

Statistical analyses, adjusting for multiple comparisons, revealed that groups were comparable on age (*U* > 37.00; *p* > 0.141), years of education (*U* > 61.00, *p* = 1.000), and duration of treatment (*U* > 47.00, *p* = 1.000). RHD had a significantly longer time since illness onset vs. OIC (*U* = 17.00, *p* = 0.024) but not vs. LHD (*U* = 46.50, *p* = 0.411). The IB-ADL scores were comparable between LHD and RHD groups (*U* = 58.00, *p* = 1.000). The ASRS scores were significantly lower in LHD vs. RHD (*U* = 24.00, *p* < 0.001).

Also, four LHD vs. zero RHD patients showed hemiparesis/hemiplegia of the dominant hand. Three RHD participants revealed unilateral inattention of the left side. In LHD and RHD participants, neuroimaging data primarily revealed brain damage in frontal, temporal, and parietal areas (LHD *n* = 5; RHD *n* = 8). One LHD patient showed multifocal vasogenic brain damage, and one RHD patient had vasogenic leukoaraiosis in the frontal lobe. From a total of 54 recruited participants, 4 patients were excluded from the final analyses due to missing data.

### Narrative Discourse Formal Aspects and Inter-Rater Reliability

All participants were capable of producing sufficient, adequate, and meaningful narrative discourse. Narratives varied in length between 10 and 402 propositions with a median of 32.89 in LHD and 63.75 in RHD.

The reliability of inter-rater agreement on categorization of thematic content and story types was very high. In detail, there was no disagreement between the raters regarding the thematic content of narratives. Non-significant between-rater differences appeared on the categorization of story types into restitution (Kendall *W* = 0.970, ChiSq = 44.622, df = 24, and *p* = 0.004), quest (Kendall *W* = 0.976, ChiSq = 44.898, df = 24, and *p* = 0.004), or chaos (Kendall *W* = 0.985, ChiSq = 45.293, df = 24, and *p* = 0.004).

### Thematic Content

The following list presents the main topics and the median (Mdn) number of utterances that were identified for each of them in the LHD and RHD groups in the current research:

1. *Medical* – medical description of the illness, treatment, and early symptoms of illness onset (LHD Mdn = 10.00; RHD Mdn = 8.33 utterances);
2. *Physical* – physical restrictions and/or improvement in this respect (LHD Mdn = 8.00; RHD Mdn = 6.17 utterances);
3. *Other* – kinship, personal world view, history of the world (LHD Mdn = 5.00; RHD Mdn = 8.00 utterances);
4. *Emotional* – subjectively perceived emotional reactivity, regulation, and expression and character changes (LHD Mdn = 5.00; RHD Mdn = 3.00 utterances);
5. *Cognitive* – subjectively perceived higher cognitive process deficits and/or improvement in this respect (LHD Mdn = 4.00; RHD Mdn = 1.57 utterances);
6. *Circumstances of illness onset* (LHD Mdn = 3.50; RHD Mdn = 3.33 utterances);
7. *Strategies of coping with the illness* (LHD Mdn = 3.00; RHD Mdn = 2.00 utterances).
8. *Interpersonal* – subjectively perceived social interaction, living conditions, and work situation change (LHD Mdn = 2.00; RHD Mdn = 4.67 utterances);
9. *Subjective theories of illness* – cognitive constructions of illness nature and cause (LHD Mdn = 2.00; RHD Mdn = 3.14 utterances).

LHD group produced significantly more utterances on cognitive ailments (*U* = 9.500, *p* = 0.015) and significantly fewer utterances on interpersonal changes (*U* = 9.000, *p* = 0.019) than RHD. Examples of utterances on the main topics are presented in Supplementary Appendix 1.

### Story Types

Participants from the LHD and RHD groups used all three types of stories due to Frank’s (1995) typology in their narratives. However, LHD used significantly less chaos story type than RHD (*U* = 32.000, *p* = 0.033; Table 2). Sample utterances of each story type are given in Supplementary Appendix 2.

**TABLE 2:** Story types percentage in narratives of LHD and RHD groups.

Story type	LHD	RHD	LHD – RHD *U* (uncorrected *p*-value)
Restitution%, Mdn	52.16	26.18	62.00 (0.903)
Quest%, Mdn	45.06	59.36	50.00 (0.391)
Chaos%, Mdn	1.12	2.39	32.000 (0.033)

*Note. LHD, left-hemisphere brain damaged patients; RHD, right-hemisphere brain damaged patients. The differences among the two groups were analyzed with Mann Whitney U test. The values present% of story types in narratives of LHD and RHD groups. The presented p-values are uncorrected for multiple comparisons.*

Left hemisphere damage patients mostly used restitution story. They talked about medical diagnosis; treatment and attempts to improve their own condition; doctor competence; being under doctors’ orders; outer locus of control; satisfaction of getting better; the goal is to get back to health; will to get back to “just before the illness onset”; illness as not their “normal state,” but an aberration of normal passage; health as a resolution of present problems (due to illness) and/or the necessity to get back to health as a primary condition of further functioning; importance of health. In terms of quest story LHD individuals mentioned: departure and early symptoms; initiation and marked return; memoir; personal experience of the illness (i.e., symptoms, changes, and deficit acceptance); illness as inspiration; alternative ways of being ill and the use of illness; and “something is gained” due to being ill (i.e., changes in attitudes/values, new social contacts). LHD participants used chaos story type in terms of repetition of the phrase “and then,” use of silences, the view of never getting better, depersonalization, and chaotic structure.

The RHD group revealed a tendency for quest story rather than restitution with a rare use of chaos story type. Quest story type characteristics of narratives in RHD patients were departure and early symptoms, initiation, and/or marked return; accommodation to new situation; memoir and manifesto; personal experience of the illness (i.e., symptoms, changes, and deficit acceptance); dyadic and/or ethic of recollection; display of character; alternative ways of being ill and/or the use of illness; “something is gained” due to being ill (i.e., changes in attitudes/values, new social contacts); and comparison of the self to the great personalities. RHD participants used restitution story in a significantly lower percentage than quest story with the following: medical diagnosis; treatment and attempts to improve their condition; doctor competence; being under doctors’ advice/care/orders; outer locus of control (i.e., doctors, God, and family) and dependence on others and responsibility limited to following treatment and taking medications; improvement in gaining health and/or satisfaction from it; illness as not their “normal state” and/or as an interruption of “the normal passage”; time before illness presented as positive, after illness as negative, but future in good health again as positive once more, or just optimistic view of getting back to health in the future; health as a resolution of present problems. The RHD group presented several characteristics of chaos story: emotional battering/suffering, anxiety and/or fear of re-experiencing illness, illness as uncontrollable/incurable, treatment as resulting in further problems, lack of support from others, depersonalization in terms of use of the form “it” for the part of the body, pessimistic view of future and/or present situation of being ill as never to change, and no correspondence to either past or future (situation of illness as the only one mentioned). RHD individuals showed the following narrative structure in chaos story type: chaotic with the use of silences and with constant repetition of the phrase “and then.”

### Cognitive Function

Cognitive assessment outcomes (i.e., raw scores) in LHD, RHD, and OIC groups are shown in Table 3. Between-group comparisons with Bonferroni-adjusted significance reveal that LHD participants showed lower scores on attention and psychomotor speed (TMT-A, *U* = 9.00, and *p* < 0.001) as compared to OIC patients. RHD showed lower scores than OIC on the following cognitive functions: abstract thinking [WAIS-R(PL), *U* = 76.50, and *p* = 0.018; RHLB-PL Inference Test, *U* = 85.00, and *p* = 0.008; RHLB-PL Picture Metaphors Test, *U* = 47.50, and *p* < 0.001], attention and psychomotor speed (TMT-A, *p* < 0.001), executive functions (WCST Percentage Errors, *U* = 6.00, and *p* = 0.009; Percentage Perseveration Errors, *U* = 10.00, and *p* = 0.024; and Percentage Conceptual Answers, *U* = 8.00, and *p* = 0.015), language functions (RHLB-PL Lexical-Semantic Test, *U* = 71.50, and *p* = 0.003; Emotional Prosody Test, *U* = 83.50, and *p* = 0.018), memory and verbal learning (CVLT A1-A5, *U* = 101.50, and *p* = 0.048; Free Recall Short Delay, *U* = 98.50, and *p* = 0.036), and visuo-spatial functions [WAIS-R(PL) Visual Puzzles, *U* = 24.50, and *p* < 0.001]. Comparisons between the two clinical groups revealed that RHD performed significantly lower on the test of visuo-spatial functions than LHD [WAIS-R(PL) Visual Puzzles, *U* = 9.00, and *p* = 0.009]. RHD also received lower scores on one of the measures of language functions (i.e., RHLB-PL Lexical-Semantic Test) than LHD; however, the difference was at the level of statistical trend (*U* = 29.50, *p* = 0.057).

**TABLE 3:** Cognitive performance in LHD, RHD, and OIC groups.

		Brain hemisphere damage					
		Left (*n* = 9) Mdn	Right (*n* = 16) Mdn	OIC (*n* = 25) Mdn	LHD – OIC *U* (Bonferroni corrected *p*-value)	RHD – OIC *U* (Bonferroni corrected *p*-value)	LHD – RHD *U* (Bonferroni corrected *p*-value)
COGNITIVE FUNCTION:	COGNITIVE TESTS (RAW OUTCOME SCORES):						
Abstract thinking and non-verbal abilities engaged in analyzing language and non-verbal data	WAIS-R(PL) – Similarities	18.00	11.00	16.50	72.50 (1.000)	**76.50 (0.018)**	28.50 (0.120)
	RHLB-PL – Inference test	14.00	13.00	15.00	65.50 (0.171)	**85.00 (0.009)**	47.50 (0.666)
	– Picture metaphors test	8.00	5.00	10.00	70.00 (0.213)	**47.50 (<0.001)**	34.00 (0.126)
Attention and psychomotor speed	TMT-A – time (sec)	80.00	64.00	34.50	**9.00 (<0.001)**	**20.50 (<0.001)**	46.50 (1.000)
Executive functions	WCST – Number of correct answers	62.00	61.50	65.50	44.50 (1.000)	2.00 (1.000)	18.00 (1.000)
	– Percent of mistakes	18.18	51.79	23.65	46.00 (1.000)	**6.00 (0.009)**	8.00 (0.189)
	– Percent of perseveration mistakes	10.81	32.92	14.34	43.00 (1.000)	**10.00 (0.024)**	7.00 (0.138)
	– Percent of conceptual answers	75.00	29.78	70.24	47.00 (1.000)	**8.00 (0.015)**	7.00 (0.138)
Language functions and understanding emotional aspect of auditory data	WAIS-R(PL) – Comprehension	16.00	13.00	17.50	68.50 (1.000)	99.00 (0.608)	44.50 (1.000)
	RHLB-PL – Lexical-semantic test	13.00	10.00	13.00	112.00 (1.000)	**71.50 (0.003)**	29.50 (0.057)
	– Emotional prosody test	13.00	11.00	13.00	107.00 (1.000)	**83.50 (0.018)**	28.00 (0.078)
Memory and verbal learning	WAIS-R(PL) – Digit span	9.00	9.00	11.50	54.50 (0.760)	107.50 (1.000)	57.50 (1.000)
	CVLT – sum A1-A5	44.00	42.00	49.00	54.00 (0.066)	**101.50 (0.048)**	60.00 (1.000)
	– Free recall after short delay	8.00	7.00	10.00	72.50 (0.339)	**98.50 (0.036)**	55.00 (1.000)
	– Free recall after long delay	7.00	9.00	9.00	86.50 (1.000)	173.00 (1.000)	51.00 (1.000)
	– Recognition	14.00	13.00	16.00	83.00 (0.651)	119.50 (0.138)	53.50 (1.000)
Visuo-spatial functions	WAIS-R(PL) – Visual puzzles	30.00	17.00	31.50	85.00 (1.000)	**24.50 (<0.001)**	**9.00 (0.009)**

*Note. LHD, left-hemisphere damage patients; RHD, right-hemisphere damage patients; OIC, orthopedic injury comparators; n, number of subjects; and Mdn, median. The Mdn values for the Cognitive Components scores are shown as the Z-scores (standardization for the mean and standard deviation of the control group). The Mdn values for the Cognitive Tests outcome scores are shown as raw, non-standardized scores. The differences among the three groups were analyzed with Mann Whitney U test. The presented p-values are Bonferroni-adjusted for multiple comparisons. Values that are statistically significant are in bold.*

### Relationship Between Thematic Content and Cognitive Functioning

After correcting for multiple comparisons, the number of utterances on particular topics was not significantly related to scores on cognitive tests in neither LHD nor RHD participants (i.e., Bonferroni-adjusted *p*-values > 0.05). However, the Spearman’s *Rho* results without Bonferroni correction indicated relationships described in the following paragraphs and visualized in Supplementary Figure 1.

In LHD participants, a higher number of utterances produced on physical difficulties following stroke was related to lower executive functions (i.e., WCST Percentage of Errors, *Rho* = 0.757, and *p* = 0.049; Percentage Conceptual Answers *Rho* = −0.757, and *p* = 0.049) and lower scores on one of the indices of memory and verbal learning (CVLT Free Recall Long Delay, *Rho* = −0.671, and *p* = 0.048; Table 4A). More utterances produced on cognitive symptoms following stroke were related to lower scores on one of the indices of executive functions (i.e., WCST Percentage of Errors, *Rho* = 0.928, and *p* = 0.008). More utterances produced on strategies of coping with the illness were linked to poorer executive functioning (WCST Percentage of Errors, *Rho* = 0.975, and *p* = 0.005; Percentage of Conceptual Answers, *Rho* = −0.975, and *p* = 0.005; and a statistical trend on Percentage of Perseveration Errors, *Rho* = 0.872, and *p* = 0.054), as well as lower memory and verbal learning (CVLT Free Recall Short Delay, *Rho* = −0.921, and *p* = 0.026; and a statistical trend on Free Recall Long Delay, *Rho* = −0.872, and *p* = 0.054). More utterances on interpersonal matters were related to lower scores on one of the indices of executive functions at a level of statistical trend (i.e., WCST Percent of Conceptual Answers, *Rho* = −0.949, and *p* = 0.051). There was a relationship between a higher number of utterances on other topics and lower scores on two indices of language functions at a level of statistical trend (RHLB-PL Lexical-Semantical Test, *Rho* = −0.949, and *p* = 0.051; Emotional Prosody Test, *Rho* = −0.949, and *p* = 0.051). There was no significant link between the number of utterances on the medical topic or circumstances of illness onset topic and cognitive tests’ outcome scores in LHD participants. The relationship was not assessed between emotional or STOI topics and cognitive tests’ outcome scores due to low variability of the number of utterances produced on these two topics in the LHD group.

**TABLE 4A:** Relationship between the thematic content and the cognitive functions (raw test outcome scores) in LHD.

		Medical, Rho (*p*)	Physical, Rho (*p*)	Other, Rho (*p*)	Emotional, Rho (*p*)	Cognitive, Rho (*p*)	Circumstances of illness onset, Rho (*p*)	Strategies of coping with the illness, Rho (*p*)	Interpersonal, Rho (*p*)	Subjective theories of illness, Rho (*p*)
Abstract thinking	WAIS-R(PL) Similarities	−0.171 (0.686)	−0.515 (0.191)	−0.632 (0.368)	−	−0.216 (0.682)	0.179 (0.701)	−0.026 (0.966)	0.224 (0.718)	–
	RHLB-PL Inference test	0.230 (0.552)	−0.142 (0.716)	−0.800 (0.200)	–	0.113 (0.809)	0.056 (0.895)	−0.564 (0.322)	−0.229 (0.710)	–
	Picture metaphors test	0.449 (0.226)	−0.087 (0.824)	−0.200 (0.800)	–	0.019 (0.967)	0.229 (0.585)	−0.658 (0.227)	0.000 (1.000)	–
Attention and Psycho-motor speed	TMT-A	0.286 (0.535)	0.432 (0.333)	0.500 (0.667)	–	0.564 (0.322)	−0.256 (0.579)	0.205 (0.741)	0.112 (0.858)	–
Executive functions	WCST Correct answers	0.071 (0.879)	−0.036 (0.939)	0.000 (1.000)	–	−0.174 (0.742)	0.030 (0.954)	−0.821 (0.089)	−0.632 (0.368)	–
	% Errors	0.607 (0.148)	**0.757 (0.049)**	0.000 (1.000)	–	**0.928 (0.008)**	0.091 (0.864)	**0.975 (0.005)**	−0.316 (0.684)	–
	% Perseveration errors	0.286 (0.535)	0.649 (0.115)	0.500 (0.667)	–	0.783 (0.066)	−0.334 (0.518)	**0.872 (0.054)**	0.738 (0.262)	–
	% Conceptual answers	−0.607 (0.148)	−**0.757 (0.049)**	0.000 (1.000)	–	−0.162 (0.759)	0.030 (0.954)	−**0.975 (0.005)**	−**0.949 (0.051)**	–
Language functions	WAIS-R(PL) Comprehension	−0.132 (0.756)	−0.572 (0.138)	−0.632 (0.368)	–	0.000 (1.000)	0.099 (0.834)	0.154 (0.805)	0.224 (0.718)	–
	RHLB-PL Lexical-semantic test	−0.465 (0.207)	−0.349 (0.357)	−**0.949 (0.051)**	–	−0.416 (0.353)	−0.063 (0.883)	−0.918 (0.028)	0.000 (1.000)	–
	Emotional prosody	−0.291 (0.448)	0.030 (0.939)	−**0.949 (0.051)**	–	−0.094 (0.841)	−0.458 (0.253)	−0.410 (0.493)	−0.229 (0.710)	–
Memory and learning	WAIS-R(PL) Digit span	0.277 (0.470)	−0.025 (0.948)	−0.600 (0.400)	–	−0.094 (0.841)	0.288 (0.489)	−0.564 (0.322)	−0.224 (0.718)	–
	CVLT A1-5	0.226 (0.559)	−0.122 (0.754)	0.000 (1.000)	–	0.259 (0.574)	−0.672 (0.068)	−0.462 (0.434)	0.335 (0.581)	–
	Free recall short delay	0.185 (0.634)	−0.068 (0.862)	0.000 (1.000)	–	0.113 (0.809)	−0.014 (0.974)	−**0.921 (0.026)**	−0.574 (0.312)	–
	Free recall long delay	0.244 (0.527)	−**0.671 (0.048)**	0.632 (0.368)	–	0.327 (0.474)	−0.084 (0.844)	−**0.872 (0.054)**	−0.335 (0.581)	–
	Recognition	−0.103 (0.793)	−0.500 (0.170)	0.738 (0.262)	–	0.132 (0.778)	0.000 (1.000)	−0.108 (0.863)	0.000 (1.000)	–
Visuo-spatial Functions	WAIS-R(PL) Visual puzzles	0.037 (0.931)	−0.247 (0.555)	−0.316 (0.684)	–	−0.134 (0.800)	0.102 (0.827)	−0.205 (0.741)	0.287 (0.640)	–

*Note. Values presented in the table are Rho and uncorrected p-values for multiple comparisons. After applying Bonferroni adjustment for multiple comparisons, there were no significant correlations at the Bonferroni-adjusted p-value of <0.05. Low variability of data for “Emotional” and “Subjective Theories of Illness” variables did not allow to execute correlation analysis between these two variables and cognitive tests’ outcome scores in LHD group. Supplementary Figure 1 presents the visualization of the significant relationships reported in this table between story types and cognitive tests’ scores in RHD group. Values that are statistically significant are in bold.*

In RHD patients, a higher number of utterances produced on cognitive difficulties following stroke was linked to lower executive functions (WCST Number of Correct Answers, *Rho* = −0.939, and *p* = 0.005; Percentage Conceptual Answers, *Rho* = −0.926, and *p* = 0.008; Table 4B) and to higher scores on one of the indices of memory and learning at a level of statistical trend (CVLT Recognition, *Rho* = 0.650, and *p* = 0.058). Similarly, more utterances on circumstances of illness onset were related to lower executive functioning (WCST Number of Correct Answers, *Rho* = −0.857, and *p* = 0.029; Percentage of Perseveration Errors, *Rho* = 0.845, and *p* = 0.034; and Percentage of Conceptual Answers, *Rho* = −0.845, and *p* = 0.034), and to higher scores on one of the indices of memory and learning (CVLT Free Recall Long Delay, *Rho* = 0.615, and *p* = 0.044). More utterances on interpersonal issues were linked to lower language functions [WAIS-R(PL) Comprehension, *Rho* = −0.642, and *p* = 0.024] and lower performance on one of the tasks relying on memory and verbal learning (CVLT Recognition, *Rho* = −0.732, and *p* = 0.007). We also found a statistical trend for the relationship between more utterances on emotional consequences of illness and lower scores on one of the indices of executive functions (WCST Number of Correct Answers, *Rho* = −0.949, and *p* = 0.051) and lower attention and psychomotor speed (TMT-A, *Rho* = 0.662, and *p* = 0.052). There was no relationship between the number of utterances on topics related to medical care, physical ailments, coping strategies, STOI, or topics classified as *other* and cognitive test scores in the RHD group.

**TABLE 4B:** Relationship between the thematic content and the cognitive functions (raw test outcome scores) in RHD.

		Medical, Rho (*p*)	Physical, Rho (*p*)	Other, Rho (*p*)	Emotional, Rho (*p*)	Cognitive, Rho (*p*)	Circumstances of illness onset, Rho (*p*)	Strategies of coping with the illness, Rho (*p*)	Interpersonal, Rho (*p*)	Subjective theories of illness, Rho (*p*)
Abstract thinking	WAIS-R(PL) Similarities	−0.007 (0.979)	−0.080 (0.776)	−0.432 (0.161)	−0.350 (0.395)	0.000 (1.000)	−0.550 (0.079)	−0.438 (0.205)	−0.113 (0.726)	−0.196 (0.641)
	RHLB-PL Inference test	0.240 (0.388)	0.103 (0.715)	0.144 (0.656)	−0.209 (0.619)	−0.216 (0.607)	0.514 (0.106)	0.167 (0.644)	−0.252 (0.430)	0.510 (0.196)
	Picture metaphors test	−0.344 (0.209)	−0.490 (0.070)	−0.357 (0.255)	−0.063 (0.882)	0.587 (0.126)	−0.030 (0.930)	−0.216 (0.550)	−0.544 (0.067)	0.616 (0.104)
Attention and Psycho- motor speed	TMT-A	−0.152 (0.589)	−0.145 (0.607)	0.238 (0.456)	**0.662 (0.052)**	−0.234 (0.544)	0.254 (0.451)	−0.060 (0.878)	0.236 (0.461)	0.290 (0.487)
Executive functions	WCST Correct answers	0.841 (0.036)	0.522 (0.288)	−0.103 (0.870)	−**0.949 (0.051)**	−**0.939 (0.005)**	−**0.857 (0.029)**	−0.026 (0.966)	0.154 (0.805)	−0.211 (0.789)
	% Errors	−0.118 (0.662)	−0.199 (0.461)	0.233 (0.443)	0.532 (0.141)	0.431 (0.246)	0.201 (0.554)	0.341 (0.334)	0.208 (0.494)	−0.039 (0.921)
	% Perseveration errors	−0.600 (0.208)	−0.500 (0.312)	−0.100 (0.873)	0.800 (0.200)	0.617 (0.192)	**0.845 (0.034)**	−0.154 (0.805)	0.000 (1.000)	0.211 (0.789)
	% Conceptual answers	0.771 (0.072	0.412 (0.417)	−0.300 (0.624)	0.000 (1.000)	−**0.926 (0.008)**	−**0.845 (0.034)**	−0.205 (0.741)	0.000 (1.000)	−0.211 (0.789)
Language functions	WAIS-R(PL) Comprehension	−0.113 (0.688)	−0.117 (0.677)	−0.507 (0.092)	−0.522 (0.185)	−0.18 (0.666)	−0.080 (0.815)	−0.476 (0.164)	−**0.642 (0.024)**	0.111 (0.794)
	RHLB-PL Lexical-semantic test	0.186 (0.507)	0.131 (0.643)	−0.394 (0.205)	−0.531 (0.176)	0.386 (0.345)	−0.370 (0.262)	−0.412 (0.236)	−0.448 (0.145)	0.299 (0.472)
	Emotional prosody	0.264 (0.362)	0.224 (0.441)	−0.041 (0.899)	−0.683 (0.062)	−0.092 (0.829)	−0.433 (0.183)	0.344 (0.331)	0.292 (0.356)	−0.419 (0.302)
Memory and learning	WAIS-R(PL) Digit span	−0.006 (0.982)	−0.168 (0.549)	−0.193 (0.548)	0.325 (0.432)	0.508 (0.199)	−0.310 (0.353)	0.019 (0.959)	0.128 (0.692)	0.291 (0.485)
	CVLT A1-5	−0.114 (0.685)	−0.159 (0.572)	0.014 (0.965)	−0.596 (0.090)	−0.106 (0.785)	0.125 (0.715)	0.145 (0.710)	0.118 (0.715)	−0.667 (0.071)
	Free recall short delay	−0.150 (0.595)	−0.402 (0.138)	−0.480 (0.114)	−0.430 (0.247)	0.464 (0.208)	0.157 (0.644)	−0.442 (0.234)	−0.516 (0.086)	−0.038 (0.929)
	Free recall long delay	0.197 (0.482)	−0.336 (0.222)	−0.025 (0.938)	−0.207 (0.593)	0.019 (0.961)	**0.615 (0.044)**	0.061 (0.876)	−0.265 (0.404)	−0.168 (0.691)
	Recognition	0.054 (0.848)	−0.312 (0.258)	−0.420 (0.174)	−0.224 (0.562)	**0.650 (0.058)**	0.106 (0.756)	−0.320 (0.401)	−**0.732 (0.007)**	0.101 (0.812)
Visuo-spatial functions	WAIS-R(PL) Visual puzzles	0.512 (0.089)	0.324 (0.304)	−0.339 (0.339)	−0.308 (0.553)	−0.359 (0.485)	−0.556 (0.120)	−0.248 (0.520)	−0.106 (0.770)	−0.241 (0.602)

*Note. Values presented in the table are Rho and uncorrected p-values for multiple comparisons. After applying Bonferroni adjustment for multiple comparisons, there were no significant correlations at the Bonferroni-adjusted p-value of <0.05. Supplementary Figure 1 presents the visualization of the significant relationships reported in this table between story types and cognitive tests’ scores in RHD group. Values that are statistically significant are in bold.*

### Relationship Between Story Types and Cognitive Functioning

After correcting for multiple comparisons, the use of each story type was not significantly related to scores on cognitive tests in either LHD or RHD participants (i.e., Bonferroni-adjusted *p*-values > 0.05). This section presents the outcomes of Spearman’s *Rho* correlations without Bonferroni correction.

In the LHD group, there was no significant relationship between quest or restitution story types and cognitive test scores (Table 5A). Low variability in the proportion of chaos story type in the narrative discourse of LHD participants did not allow performing correlation analysis between this story type and cognitive tests’ scores.

**TABLE 5A:** Relationship between the story type and the cognitive functions (raw test outcome scores) in LHD.

		Quest story type (%), Rho (*p*)	Restitution story type (%), Rho (*p*)	Chaos story type (%), Rho (*p*)
Abstract thinking	WAIS-R(PL) – Similarities	0.146 (0.729)	−0.466 (0.244)	–
	RHLB-PL – Inference test	−0.108 (0.798)	0.279 (0.504)	–
	– Picture metaphors test	−0.282 (0.498)	0.185 (0.661)	–
Attention and Psycho- motor speed	TMT-A	−0.321 (0.482)	0.378 (0.403)	–
Executive functions	WCST – Correct answers	0.107 (0.819)	0.107 (0.819)	–
	– % Errors	−0.107 (0.819)	0.607 (0.148)	–
	– % Perseveration errors	−0.214 (0.645)	−0.036 (0.939)	–
	– % Conceptual answers	0.107 (0.819)	−0.607 (0.148)	–
Language functions	WAIS-R(PL) – Comprehension	0.072 (0.866)	−0.386 (0.346)	–
	RHLB-PL – Lexical-semantic test	0.047 (0.912)	−0.298 (0.474)	–
	– Emotional prosody	0.417 (0.304)	0.117 (0.782)	–
Memory and learning	WAIS-R(PL) – Digit span	0.084 (0.844)	0.373 (0.362)	–
	CVLT – A1-5	−0.563 (0.146)	0.223 (0.596)	–
	– Free recall short delay	−0.108 (0.798)	0.285 (0.494)	–
	– Free recall long delay	−0.342 (0.408)	0.663 (0.073)	–
	– Recognition	−0.321 (0.438)	0.075 (0.861)	–
Visuo-spatial functions	WAIS-R(PL) – Visual puzzles	−0.089 (0.840)	0.185 (0.661)	–

*Note. Values presented in the table are Rho and uncorrected p-values for multiple comparisons. After applying Bonferroni adjustment for multiple comparisons, there were no significant correlations at the Bonferroni-adjusted p-value of < 0.05. Low variability of data for “Chaos story type” variable did not allow to execute correlation analysis between this variable and cognitive tests’ outcome scores in LHD group.*

In the RHD group, more frequent use of the chaos story type was linked to lower scores on indices of executive functioning (WCST Number of Correct Answers, *Rho* = −0.893, and *p* = 0.016; Percentage of Conceptual Answers, *Rho* = −0.880, and *p* = 0.021; Table 5B; Supplementary Figure 1). There was no relationship between quest or restitution story types and cognitive test scores in the RHD group.

**TABLE 5B:** Relationship between the story type and the cognitive functions (raw test outcome scores) in RHD.

		Quest story type (%), Rho (*p*)	Restitution story type (%), Rho (*p*)	Chaos story type (%), Rho (*p*)
Abstract thinking	WAIS-R(PL) – Similarities	0.247 (0.374)	−0.073 (0.797)	−0.244 (0.380)
	RHLB-PL – Inference test	0.184 (0.512)	−0.285 (0.303)	0.039 (0.889)
	– Picture metaphors test	−0.353 (0.197)	−0.072 (0.799)	0.361 (0.186)
Attention and Psycho- motor speed	TMT-A	−0.113 (0.688)	0.030 (0.914)	0.157 (0.576)
Executive functions	WCST – Correct answers	0.551 (0.257)	0.725 (0.103)	−**0.893 (0.016)**
	– % Errors	0.289 (0.278)	−0.275 (0.302)	0.275 (0.303)
	– % Perseveration errors	−0.714 (0.111)	−0.143 (0.787)	0.516 (0.295)
	– % Conceptual answers	0.429 (0.397)	0.771 (0.072)	−**0.880 (0.021)**
Language functions	WAIS-R(PL) – Comprehension	0.084 (0.765)	−0.084 (0.765)	−0.207 (0.460)
	RHLB-PL – Lexical-semantic test	−0.305 (0.268)	0.154 (0.585)	0.143 (0.610)
	– Emotional prosody	0.133 (0.650)	0.053 (0.858)	−0.063 (0.830)
Memory and learning	WAIS-R(PL) – Digit span	0.128 (0.649)	−0.172 (0.541)	0.160 (0.569)
	CVLT – A1-5	0.181 (0.520)	−0.102 (0.718)	0.106 (0.708)
	– Free recall short delay	0.020 (0.944)	−0.087 (0.758)	0.063 (0.824)
	– Free recall long delay	0.049 (0.862)	−0.040 (0.888)	−0.044 (0.875)
	– Recognition	−0.067 (0.813)	−0.078 (0.784)	0.130 (0.643)
Visuo-spatial functions	WAIS-R(PL) – Visual puzzles	0.159 (0.622)	0.152 (0.637)	−0.435 (0.157)

*Note. Values presented in the table are Rho and uncorrected p-values for multiple comparisons. After applying Bonferroni adjustment for multiple comparisons, there were no significant correlations at the Bonferroni-adjusted p-value of < 0.05. Supplementary Figure 1 presents the visualization of the significant relationships between story types and cognitive tests’ scores in RHD group. Values that are statistically significant are in bold.*

## Discussion

### Narrative Discourse Analysis as a Source of Additional Information on Cognitive Functioning in Stroke Patients

This study provides preliminary evidence that a stroke survivor’s narrative can constitute an additional source of clinically relevant information regarding patient’s experience of illness and attitude toward recovery. Our data show that this knowledge can be unfolded with the use of discourse analyses methodologies such as the thematic framework of illness narratives (Pluta et al., 2015) and the story typology (Frank, 1995). We find that both the thematic content and the story types (i.e., restitution, quest, and chaos) are related to the severity of cognitive deficits in stroke patients who experienced left or right hemisphere damage.

The extent to which LHD patients elaborate on strategies of coping with the illness, physical or cognitive difficulties, and interpersonal matters is positively related to more profound disturbances in executive functions. Also, those patients who talk more about their coping strategies and physical ailments show lower abilities to recall memorized verbal information after a delay. However, elaboration on any of the identified topics is not related to performance on cognitive tests that primarily examine abstract thinking beyond the information given in the exercise, attention and psychomotor speed, or visuo-spatial functions in LHD patients. There is also no relationship between the use of quest or restitution story type and the level of cognitive functioning in LHD patients. Small sample size might have contributed to the observed low variability in the use of chaos story type within the group of LHD patients and limited our capacity to examine the relationship between this story type and performance on cognitive measures.

Right hemisphere damage patients who elaborate more on cognitive disturbances following stroke and on circumstances of illness onset also show more profound decline in executive functions. Patients who talk more about interpersonal issues reveal decline in particular aspects of language skills, such as verbal reasoning/conceptualization and verbal comprehension/expression, as well as retrieval of verbal information from memory. The extent of elaborating on topics related to medical care, emotional issues, physical ailments, coping strategies, or subjective theories of illness are not linked to the level of any of the examined cognitive functions. RHD patients who use more chaos story type in their narrative exhibit deterioration in executive functions. Higher use of quest or restitution story types is not related to cognitive functioning in RHD patients.

Together, the above findings highlight that clinicians may be alarmed when patients disclose their concern, especially regarding cognitive functioning following stroke, as those may be signs of more profound cognitive difficulties, primarily in executive functions. Additionally, elaboration on coping strategies in the case of LHD patients and circumstances of illness onset as well as interpersonal issues in RHD patients may be further linked to difficulties in memory or language functions. Furthermore, the use of chaos story type is related to executive function in RHD patients. Stroke in the RHD can lead to emotional dysregulation (Binder, 1984), which could ground more emotional turmoil expressed with the more frequent use of the chaos story type. Stroke survivors with RHD damage show deficits with emotional recognition, which can contribute to increased complaints of frustration or social isolation (Yuvaraj et al., 2013). In line with the literature, narrations of our participants with RHD damage revealed more preoccupation with interpersonal issues or emotional turmoil than participants after left brain hemisphere damage. It is possible that our RHD participants had increased emotional liability as a result of the brain damage caused by stroke. However, this study did not include measures of emotional functions. Thus, it cannot be ruled out that our RHD patients suffered from emotional deficits with neurological underpinnings. Also, the current discussion is based on the interpretation of statistical results uncorrected for multiple comparisons. The above results lost statistical significance after correcting for multiple comparisons with Bonferroni-adjusted significance. Due to these limitations, we cannot draw conclusions on whether discourse analysis methods can aid in estimation of cognitive function in stroke survivors with right vs. left brain hemisphere damage. Therefore, the currently discussed results should be treated with caution and confirmed in future research in a large sample of stroke survivors.

### Characteristics of Thematic Content of Narrative Discourse in Stroke Survivors

With the use of the thematic framework of illness narratives (Pluta et al., 2015), we show that stroke patients report on their cognitive impairment, especially in language functions. However, cognitive changes were not the main theme in the narrations of our participants as suggested in previous studies (Hjelmblink and Holmstrom, 2006). This inconsistency may be partially explained by the different severity of physical symptoms between the studied cohorts. Our participants were less physically independent than in the study by Hjelmblink and Holmstrom (2006). It is possible that our patients elaborated more on the visible physical disabilities as compared to participants in other studies who were more physically capable. Furthermore, some stroke survivors may have limited awareness of their post-stroke cognitive disabilities (Barrett et al., 2014), which can contribute to limited elaboration on cognitive challenges. Instead, *medical treatment* and *physical ailments* occupied almost half of our participants’ narrations. Utterances on treatment could be alternatively categorized as *active coping* and interpreted as an attempt of the patient to escape or reduce the stressor or its effects (after Carver et al., 1989). However, in the current study, this theme was called *medical treatment* as our participants used professional medical nomenclature and precise descriptions of treatment procedures. For example, “They had this method there, thrombolysis, and they were treating me with this method” (P.E., 53 years old, RHD). Other main themes included *emotional changes caused by illness*, c*ircumstances of illness onset, strategies of coping with the illness, and interpersonal relations* consistent with previous literature (Prigatano, 1999; McKevitt, 2000; Hjelmblink and Holmstrom, 2006; Gibson and Watkins, 2011; van der Riet et al., 2011a; Jones and Morris, 2012; Nasr et al., 2016). *Irrelevant topics* were also incorporated in patients’ narratives as previously described with such detours in the narrations pointing to the complexity of the illness experience (van der Riet et al., 2011a).

### Characteristics of Story Types Used in Narrative Discourse by Stroke Survivors

Stroke survivors typically incorporate all three types of stories (i.e., quest, restitution, and chaos) in line with previous findings in the general population (Frank, 1995). Our analysis shows that restitution (52%) and quest (45%) story characteristics occupied the majority of narratives in LHD participants, while RHD patients used more quest (59%) vs. restitution (26%) story type. Chaos story type was the most seldom and present in 1.5% and 2.5% of utterances in LHD and RHD, respectively, similarly to previous findings in stroke patients (France et al., 2013).

Restitution story was characterized by describing illness as a disruption in the normal life passage. Our patients expressed that they wished to get back to health or the moment just before the illness onset, which adds to the literature showing that patients may experience an autobiographical disruption and a temporal split due to illness (Hjelmblink and Holmstrom, 2006). Our participants also separate their “sick” body from rather intact “self” as previously shown (van der Riet et al., 2011a). Patients further highlight how thankful they are to the professionals for the progress in their rehabilitation, which resembles earlier research (van der Riet et al., 2011a).

Quest story was primarily characterized in our participants by the acceptance of the current state. Patients highlight that they do “not look back”; instead they acknowledge that disease and disability can be expected with age and appreciate being able to perform “old” activities in a “new” way, which confirms other studies (France et al., 2013). They further describe looking for alternative use of the illness in order to profit from their situation. Previous studies showed similar results in TIA patients, who describe it as a fortunate warning or opportunity to engage in more healthy behaviors to reduce the risk of stroke in the future (Gibson and Watkins, 2011).

Chaos story characteristics often make the narrative challenging to follow as patient voices seem to be mixed or conflicting (van der Riet et al., 2011b), which is confirmed in our findings. Our participants noted emotional battering, no hope for life ever getting better, and expressed being left alone and uncared for, which confirms previous reports (Jones and Morris, 2012). In former studies, patients also reported existential problems, vulnerability, and finitude; expressed hopelessness, fragility, fear, and depression (van der Riet et al., 2011b); or showed uncertainty and anxiety about the future (Hjelmblink and Holmstrom, 2006).

It should be noted that the task of assigning utterances to story type is complex and subjective. Our inter-rater analyses show that this is especially true in the case of determining between quest and restitution stories, and chaos stories are more distinctive and easier to differentiate. Importantly, our data show that it is the presence of the chaos story type that is linked to the severity of cognitive impairment. Thus, what seems especially important from the clinical perspective is the ability to pick up whether the patient uses the chaos story type or not. In our participants, the structure of chaos stories was distinguishable by a frequent use of silences. Silences may communicate that the patient is resistant to talk, think, or ascribe any meaning to illness. Previous studies show that patients may be reluctant to produce discourse on their illness or even engage in a “creative process of ordering, contemplation, and evocation in general” (McKevitt, 2000). Authors of the mentioned study suggest that such resistance may have cultural underpinnings. Although none of our participants refused to narrate or being recorded, the use of chaos story type may still be an expression of reluctance to engage in the processes of overtly analyzing their own illness experiences.

### Diagnostic Utility of Narrative Discourse Analysis Approach in Stroke Patients

The current results suggest that the methods of narrative analyses may help to draw diagnostic hypothesis based on what difficulties the patient reports (i.e., thematic framework method) as well as the way the patient talks about their experiences (i.e., story type). These methods can become an especially powerful clinical tool in raising red flags about cognitive impairment in patients who have limited capacity to perform or adhere to the standard testing protocol. They also provide additional information about the approach of patients toward their own situation and can help determine when patients should undergo mental health testing for depression, anxiety, or post-traumatic stress disorder. These are common mental health issues in stroke survivors (Kneebone and Lincoln, 2012), and early diagnosis and treatment of these issues can significantly improve health outcomes (Kneebone and Lincoln, 2012). Still, further research using a large sample should precede clinical application of the current methodology.

Evidence of the significant link between thematic content, story type, and severity of cognitive deficits further facilitates the use of patient narrative by nurse practitioners. Being at the frontier of patient care, nurses are the primary providers of patient care on everyday bases. Thus, they need to be better equipped with methods to track patient states on everyday bases (Buckley et al., 2016). Analyses of content and story type can help nurses to gain a better understanding of patient states, including cognitive difficulties (Buckley et al., 2016). We provide evidence that the thematic framework of illness narratives (Pluta et al., 2015) and story typology (Frank, 1995) can be useful tools achieving this goal. Nurses can use these methods to gain knowledge about a patient’s experience and use it to address individual patient needs, refocus or prioritize care, and alert neuropsychologists or other medical staff about potential changes in the cognitive and mental functioning of the patient. Again, it should be highlighted that, even though our results suggest a relationship between the thematic content, story type, and severity of cognitive deficits, clinical utility of the current methods should be verified with further research.

### Eliciting Discourse as a Tool for Improving Patient Care and Outcomes

Finally, it should be highlighted that eliciting discourse in stroke patients serves a dual purpose. As shown in the current study, it can aid clinicians in unfolding patients’ experiences of illness and their attitude toward current state and treatment. The proposed methods of narrative analysis equip clinicians to obtain information from the patient that is relevant to the design of individualized rehabilitation. However, promoting the production of illness narratives in stroke patients can also directly benefit health outcomes of treatment. This kind of direct impact has previously been suggested as discourse is one of the major forms of perceiving, experiencing, and evaluating one’s own actions as well as judging the course and value of one’s own life (Hyden, 1997). Production of discourse on illness may be a viable option in facilitating recovery in stroke survivors as it is a part of the narrative therapy (Chow, 2018). This “meaning-making intervention tool” is effective in improving mental health and well-being with results sustained for at least 4 months post-intervention (Chow, 2018). Thus, clinicians should aim at supporting and exploring patient’s utterances (Charon, 2011), in particular those related to subjective illness experience (Brody, 2002).

## Limitations

Limitations of this study include a relatively small sample size. Stroke diagnosis was confirmed with neuroimaging methods in our participants except for three LHD and five RHD patients. Additionally, no patients showing symptoms of aphasia were included in this research as per study design. Neuropsychologists directed only those patients to research personnel who did not show symptoms of aphasia and received high scores on the ASRS. Consequently, the ASRS scores were not accounted for in the current analyses due to the ceiling effect and insufficient variability. Thus, our findings should be treated with caution as we did not analyze patients with aphasia. Our results are relevant to stroke survivors with left hemisphere damage who do not show symptoms of aphasia as other cognitive deficits presented by participants of this study were consistent with the existing literature (e.g., Nys et al., 2005; Loetscher and Lincoln, 2013; Andrews et al., 2014; Duclos et al., 2014; Elliott and Parente, 2014; Motta et al., 2014). Furthermore, it should be noted that inter-rater reliability of the proposed methodology should be confirmed in future studies with a larger sample. There is a possibility of bias in cases in which the same professional administers the cognitive assessment and interview and rates the scripts for thematic content and story type. On the other hand, it could also be argued that being involved in data collection and the scoring/rating process provides a more in-depth understanding and allows a more accurate determination of story type, for example, by additional information related to non-verbal communication, such as facial expressions of the patient during the interview. This additional information can benefit clinical diagnostic process. It should further be noted that narrative discourse was split into utterances by one neuropsychologist in consultation with the author who originally published the methodology of thematic analysis (Pluta et al., 2015). As the current study focuses on analyzing the relationship between the thematic content, story types, and cognitive deficits, the analyses of the relationship between the formal aspects of the discourse (i.e., the number of utterances) and cognitive deficits were beyond of the scope of this study. For that reason, the inter-rater reliability was checked for categorizing utterances into themes and story types, but not for distinguishing the utterances. Lack of disagreement on the classification of utterances into particular topics poses a question about the generalization of such high inter-rater reliability. This effect can be a product of both raters having a list of proposed topics and having trained together on how to categorize utterances by topics on sample scripts (not from the current study) before attempting to categorize utterances for the current study. Also, it is possible that some minor disagreement would have appeared on the topics that were categorized as “other.” However, one rater proposed possible categories of topics that were infrequent, and the second rater categorized all infrequent topics under a common category of “other.” This discrepancy resulted from the first rater categorizing each script in turn, and the second rater reading all scripts at first and then rereading each script in turn to categorize utterances by topics. Future research should verify the inter-rater reliability and recommend raters to either rate each script in turn or familiarize themselves with all scripts up front and then read them again in order to categorize the utterances. Nonetheless, lack of disagreement between raters can also inform us that training in the thematic content analysis of narrative discourse can be helpful for neuropsychologists who are interested in using this methodology as it seems to result in high inter-rater agreement. However, the same raters also trained together before attempting to categorize the utterances by story types, and they reached a high but not 100% agreement when using this method. Together, it seems that categorization of thematic content is a more reliable method of narrative discourse than story typology. The discourse in four participants was alternating between the story types in a way that the utterances on the verge of the two story types contained characteristics of both of those story types. It was decided that these sentences should be categorized as both story types. Consequently, in four participants, the sum of percentage of discourse using restitution, quest, and chaos story types equaled more than 100%. In detail, those were four RHD patients and their total story type percentage ranged from 101.37 to 122.89%. The utterances with overlapping story types were restitution and quest story types. Another limitation is that the main outcomes of this study are interpreted based on the *p*-values uncorrected from multiple comparisons. None of the pairwise comparisons between narrative topics, story types, and cognitive component scores were significant after Bonferroni-adjustment for multiple comparisons. Due to a small sample size, it is possible that the significance of our findings failed to reach statistical significance. Also, the LHD group has fewer observations than the RHD group, which further limits the ability to find significant results. In order not to underreport potentially important findings, we additionally interpreted the statistically significant uncorrected *p*-value results. Finally, our analyses revealed that LHD and RHD groups differed in terms of cognitive abilities, which impacts the correlation analysis between cognitive tests’ scores and narrative discourse outcomes (i.e., topics and story types). Larger cohort studies are needed to confirm the current results.

## Conclusion

To sum up, this study evaluated the use of narrative discourse analyses, such as the thematic framework of illness narratives (Pluta et al., 2015) and story typology (Frank, 1995) in stroke survivors. Our findings highlight that espousing patient’s language can benefit the process of diagnosing cognitive impairment. Paying attention to the topics that the patient elaborates on as well as the story type the patient uses may help nurses and other professional staff members to get a better grasp of a patient’s difficulties, approach to illness, current situation, and treatment. This knowledge can aid everyday interactions with the patients and enable the staff to offer better care and support to the patient. Our findings show that the relationship between the thematic content, the use of particular story types and cognitive deficits is more apparent in stroke survivors with right as compared with left brain hemisphere damage. However, considering the small sample size and more observations in the former vs. latter group, these conclusions should be confirmed with further research in a larger patient cohort with left and right brain hemisphere damage.

## Data Availability Statement

The datasets presented in this article are not readily available because the datasets for this manuscript are not publicly available in order to protect anonymity of participants. Original analyzed discourse is in Polish and translation poses a risk of losing sensitive information to narrative analysis. Requests to access the datasets should be directed to AE, anna.r.egbert@gmail.com.

## Ethics Statement

The studies involving human participants were reviewed and approved by Ethics Committee of the Faculty of Psychology, University of Warsaw. The patients/participants provided their written informed consent to participate in this study.

## Author Contributions

AE conducted the assessments, prepared and analyzed data, and drafted the manuscript. All authors were involved in the study design, data analyses, interpretation, and revising the manuscript.

## Conflict of Interest

The authors declare that the research was conducted in the absence of any commercial or financial relationships that could be construed as a potential conflict of interest.
