# Supplementary material for: In Search for the Meaning of Illness: Content of Narrative Discourse Is Related to Cognitive Deficits in Stroke Patients
Source: Front Psychol. 2021 Jan 18;11:548802. doi: 10.3389/fpsyg.2020.548802 (PMC7847845; doi:10.3389/fpsyg.2020.548802)
Supplement: Supplementary file 2 [file Table_1.DOCX]

**Table 1.** Demographic and clinical characteristics of the sample.

|  | **Brain hemisphere damage** | |  |  |  |  |
| --- | --- | --- | --- | --- | --- | --- |
|  | **Left** | **Right** | **OIC** |  |  |  |
|  | (*n* = 9) | (*n* = 16) | (*n* = 25) |  |  |  |
|  | Mdn | Mdn | Mdn | **LHD – OIC**  *U* (Bonferroni corrected *p-*value) | **RHD - OIC**  *U* (Bonferroni corrected *p-*value) | **LHD - RHD**  *U* (Bonferroni corrected *p-*value) |
| Age, Mdn | 57.00 | 68.50 | 63.00 | 84.50 (0.822) | 150.50 (0.555) | 37.00 (0.141) |
| Male : female | 6 : 3 | 8 : 8 | 13 : 12 | - | - | - |
| Education (years), Mdn | 13.00 | 13.00 | 13.00 | 103.00 (1.000) | 184.00 (1.000) | 61.00 (1.000) |
| Right : left handedness | 9 : 0 | 16 : 0 | 24 : 1 | - | - | - |
| Time since illness onset (years), Mdn | 0.50 | 1.00 | 0.50 | 36.00 (.852) | 17.00 (0.024) | 46.50 (0.411) |
| Rehabilitation period (months), Mdn | 1.00 | 1.50 | 1.00 | 47.00 (1.000) | 77.00 (1.000) | 59.50 (1.000) |
| IB-ADL, Mdn | 18.00 | 17.00 | - | - | - | 58.00 (1.000) |
| ASRS, Mdn | 5.00 | 6.00 | - | - | - | 24.00 (<0.001) |
| Hemiparesis / hemiplegia of the dominant hand | 4 | 0 | - | - | - |  |
| Neuroimaging results:  - hypodensive areas in FL, TL, PL of LH  - hypodensive areas in FL, PL of LH  - hiperdensive areas in FL, TL, PL of LH  - hiperdensive areas in FL, PL, insula of LH  - hypodensive areas in FL of RH  - hypodensive areas in FL, TL, PL of RH  - hypodensive areas in TL of RH  - hypodensive areas in RH, generalized cortico-subcortical atrophy, expansion of intracranial fluid spaces  - hyperdensive areas in FL, TL, PL of RH  - hyperdensive areas in FL of RH  - multifocal vasogenic brain damage  - vasogenic leukoaraiosis in FL, PL  - not assessed | 1  2  1  1  -  -  -  -  -  -  1  -  3 | -  -  -  -  2  1  2  1  1  1  2  1  5 | -  -  -  -  -  -  -  -  -  -  -  -  25 |  |  |  |

*Note.* LHD = left-hemisphere damage patients; RHD = right-hemisphere damage patients; OIC = orthopaedic injury comparators; Mdn = median; LH = left hemisphere; RH = right hemisphere; FL = frontal lobe; TL = temporal lobe; PL = parietal lobe; ASRS = Aphasia Severity Rating Scale; IB-ADL = Barthel Index of Activities of Daily Living. The presented *p*-values are Bonferroni-adjusted for multiple comparisons.
