# Supplementary material for: In Search for the Meaning of Illness: Content of Narrative Discourse Is Related to Cognitive Deficits in Stroke Patients
Source: Front Psychol. 2021 Jan 18;11:548802. doi: 10.3389/fpsyg.2020.548802 (PMC7847845; doi:10.3389/fpsyg.2020.548802)
Supplement: Supplementary file 3 [file Table_2.DOCX]

**Table 2.** Story types percentage in narratives of LHD and RHD groups.

| Story type | LHD | RHD | LHD – RHD  *U* (uncorrected *p*-value) |
| --- | --- | --- | --- |
| Restitution %, Mdn | 52.16 | 26.18 | 62.00 (0.903) |
| Quest %, Mdn | 45.06 | 59.36 | 50.00 (0.391) |
| Chaos %, Mdn | 1.12 | 2.39 | 32.000 (0.033) |

*Note*. LHD = left-hemisphere brain damaged patients; RHD = right-hemisphere brain damaged patients. The differences among the two groups were analysed with Mann Whitney *U* test. The values present % of story types in narratives of LHD and RHD groups. The presented p-values are uncorrected for multiple comparisons.
