# Supplementary material for: In Search for the Meaning of Illness: Content of Narrative Discourse Is Related to Cognitive Deficits in Stroke Patients
Source: Front Psychol. 2021 Jan 18;11:548802. doi: 10.3389/fpsyg.2020.548802 (PMC7847845; doi:10.3389/fpsyg.2020.548802)
Supplement: Supplementary file 4 [file Table_3.DOCX]

**Table 3.** Cognitive performance in LHD, RHD, and OIC groups.

|  |  | **Brain hemisphere damage** | |  |  |  |  |
| --- | --- | --- | --- | --- | --- | --- | --- |
|  |  | **Left** | **Right** | **OIC** |  |  |  |
|  |  | (*n* = 9) | (*n* = 16) | (*n* = 25) |  |  |  |
|  |  | Mdn | Mdn | Mdn | **LHD – OIC**  *U* (Bonferroni corrected *p-*value) | **RHD - OIC**  *U* (Bonferroni corrected *p-*value) | **LHD - RHD**  *U* (Bonferroni corrected *p-*value) |
| **COGNITIVE**  **FUNCTION:** | **COGNITIVE TESTS**  **(RAW OUTCOME SCORES):** |  |  |  |  |  |  |
| Abstract thinking and non-verbal abilities engaged in analysing language and nonverbal data | WAIS-R(PL) - Similarities | 18.00 | 11.00 | 16.50 | 72.50 (1.000) | **76.50 (0.018)** | 28.50 (0.120) |
|  | RHLB-PL - Inference Test | 14.00 | 13.00 | 15.00 | 65.50 (0.171) | **85.00 (0.009)** | 47.50 (0.666) |
|  | - Picture Metaphors Test | 8.00 | 5.00 | 10.00 | 70.00 (0.213) | **47.50 (<0.001)** | 34.00 (0.126) |
| Attention and psychomotor speed | TMT-A - time (sec) | 80.00 | 64.00 | 34.50 | **9.00 (<0.001)** | **20.50 (<0.001)** | 46.50 (1.000) |
| Executive functions | WCST - Number of correct answers | 62.00 | 61.50 | 65.50 | 44.50 (1.000) | 2.00 (1.000) | 18.00 (1.000) |
|  | - Percent of mistakes | 18.18 | 51.79 | 23.65 | 46.00 (1.000) | **6.00 (0.009)** | 8.00 (0.189) |
|  | - Percent of perseveration mistakes | 10.81 | 32.92 | 14.34 | 43.00 (1.000) | **10.00 (0.024)** | 7.00 (0.138) |
|  | - Percent of conceptual answers | 75.00 | 29.78 | 70.24 | 47.00 (1.000) | **8.00 (0.015)** | 7.00 (0.138) |
| Language functions and understanding emotional aspect of auditory data | WAIS-R(PL) - Comprehension | 16.00 | 13.00 | 17.50 | 68.50 (1.000) | 99.00 (0.608) | 44.50 (1.000) |
|  | RHLB-PL - Lexical-Semantic Test | 13.00 | 10.00 | 13.00 | 112.00 (1.000) | **71.50 (0.003)** | 29.50 (0.057) |
|  | - Emotional Prosody Test | 13.00 | 11.00 | 13.00 | 107.00 (1.000) | **83.50 (0.018)** | 28.00 (0.078) |
| Memory and verbal learning | WAIS-R(PL) - Digit Span | 9.00 | 9.00 | 11.50 | 54.50 (0.760) | 107.50 (1.000) | 57.50 (1.000) |
|  | CVLT - sum A1-A5 | 44.00 | 42.00 | 49.00 | 54.00 (0.066) | **101.50 (0.048)** | 60.00 (1.000) |
|  | - Free Recall After Short Delay | 8.00 | 7.00 | 10.00 | 72.50 (0.339) | **98.50 (0.036)** | 55.00 (1.000) |
|  | - Free Recall After Long Delay | 7.00 | 9.00 | 9.00 | 86.50 (1.000) | 173.00 (1.000) | 51.00 (1.000) |
|  | - Recognition | 14.00 | 13.00 | 16.00 | 83.00 (0.651) | 119.50 (0.138) | 53.50 (1.000) |
| Visuo-spatial functions | WAIS-R(PL) - Visual Puzzles | 30.00 | 17.00 | 31.50 | 85.00 (1.000) | **24.50 (<0.001)** | **9.00 (0.009)** |

*Note*. LHD = left-hemisphere damage patients; RHD = right-hemisphere damage patients; OIC = orthopaedic injury comparators; *n* = number of subjects; Mdn = median. The Mdn values for the Cognitive Components scores are shown as the Z-scores (standardization for the mean and standard deviation of the control group). The Mdn values for the Cognitive Tests outcome scores are shown as raw, nonstandardized scores. The differences among the three groups were analysed with Mann Whitney *U* test. The presented *p*-values are Bonferroni-adjusted for multiple comparisons.
