# Supplementary material for: In Search for the Meaning of Illness: Content of Narrative Discourse Is Related to Cognitive Deficits in Stroke Patients
Source: Front Psychol. 2021 Jan 18;11:548802. doi: 10.3389/fpsyg.2020.548802 (PMC7847845; doi:10.3389/fpsyg.2020.548802)
Supplement: Supplementary file 5 [file Table_4.DOCX]

**Table 4.a.** Relationship between the thematic content and the cognitive functions (raw test outcome scores) in LHD.

|  |  | **Medical, Rho (*p*)** | **Physical, Rho (*p*)** | **Other, Rho (*p*)** | **Emotional, Rho (*p*)** | **Cognitive, Rho (*p*)** | **Circumstances of illness onset, Rho (*p*)** | **Strategies of coping with the illness, Rho (*p*)** | **Interpersonal, Rho (*p*)** | **Subjective theories of illness, Rho (*p*)** |
| --- | --- | --- | --- | --- | --- | --- | --- | --- | --- | --- |
| **Abstract Thinking** | WAIS-R(PL)  Similarities | -0.171 (0.686) | -0.515 (0.191) | -0.632 (0.368) | - | -0.216 (0.682) | 0.179 (0.701) | -0.026 (0.966) | 0.224 (0.718) | - |
|  | RHLB-PL  Inference Test | 0.230 (0.552) | -0.142 (0.716) | -0.800 (0.200) | - | 0.113 (0.809) | 0.056 (0.895) | -0.564 (0.322) | -0.229 (0.710) | - |
|  | Picture Metaphors Test | 0.449 (0.226) | -0.087 (0.824) | -0.200 (0.800) | - | 0.019 (0.967) | 0.229 (0.585) | -0.658 (0.227) | 0.000 (1.000) | - |
| **Attention and Psycho-**  **motor Speed** | TMT-A | 0.286 (0.535) | 0.432 (0.333) | 0.500 (0.667) | - | 0.564 (0.322) | -0.256 (0.579) | 0.205 (0.741) | 0.112 (0.858) | - |
| **Executive Functions** | WCST  Correct Answers | 0.071 (0.879) | -0.036 (0.939) | 0.000 (1.000) | - | -0.174 (0.742) | 0.030 (0.954) | -0.821 (0.089) | -0.632 (0.368) | - |
|  | % Errors | 0.607 (0.148) | **0.757 (0.049)** | 0.000 (1.000) | - | **0.928 (0.008)** | 0.091 (0.864) | **0.975 (0.005)** | -0.316 (0.684) | - |
|  | % Perseveration Errors | 0.286 (0.535) | 0.649 (0.115) | 0.500 (0.667) | - | 0.783 (0.066) | -0.334 (0.518) | **0.872 (0.054)** | 0.738 (0.262) | - |
|  | % Conceptual Answers | -0.607 (0.148) | **-0.757 (0.049)** | 0.000 (1.000) | - | -0.162 (0.759) | 0.030 (0.954) | **-0.975 (0.005)** | **-0.949 (0.051)** | - |
| **Language Functions** | WAIS-R(PL)  Comprehension | -0.132 (0.756) | -0.572 (0.138) | -0.632 (0.368) | - | 0.000 (1.000) | 0.099 (0.834) | 0.154 (0.805) | 0.224 (0.718) | - |
|  | RHLB-PL  Lexical-Semantic Test | -0.465 (0.207) | -0.349 (0.357) | **-0.949 (0.051)** | - | -0.416 (0.353) | -0.063 (0.883) | -0.918 (0.028) | 0.000 (1.000) | - |
|  | Emotional Prosody | -0.291 (0.448) | 0.030 (0.939) | **-0.949 (0.051)** | - | -0.094 (0.841) | -0.458 (0.253) | -0.410 (0.493) | -0.229 (0.710) | - |
| **Memory and Learning** | WAIS-R(PL)  Digit Span | 0.277 (0.470) | -0.025 (0.948) | -0.600 (0.400) | - | -0.094 (0.841) | 0.288 (0.489) | -0.564 (0.322) | -0.224 (0.718) | - |
|  | CVLT  A1-5 | 0.226 (0.559) | -0.122 (0.754) | 0.000 (1.000) | - | 0.259 (0.574) | -0.672 (0.068) | -0.462 (0.434) | 0.335 (0.581) | - |
|  | Free Recall Short Delay | 0.185 (0.634) | -0.068 (0.862) | 0.000 (1.000) | - | 0.113 (0.809) | -0.014 (0.974) | **-0.921 (0.026)** | -0.574 (0.312) | - |
|  | Free Recall Long Delay | 0.244 (0.527) | **-0.671 (0.048)** | 0.632 (0.368) | - | 0.327 (0.474) | -0.084 (0.844) | **-0.872 (0.054)** | -0.335 (0.581) | - |
|  | Recognition | -0.103 (0.793) | -0.500 (0.170) | 0.738 (0.262) | - | 0.132 (0.778) | 0.000 (1.000) | -0.108 (0.863) | 0.000 (1.000) | - |
| **Visuo-spatial Functions** | WAIS-R(PL)  Visual Puzzles | 0.037 (0.931) | -0.247 (0.555) | -0.316 (0.684) | - | -0.134 (0.800) | 0.102 (0.827) | -0.205 (0.741) | 0.287 (0.640) | - |

*Note.* Values presented in the table are Rho and uncorrected p-values for multiple comparisons. After applying Bonferroni adjustment for multiple comparisons, there were no significant correlations at the Bonferroni-adjusted *p*-value of <0.05. Low variability of data for “Emotional” and “Subjective Theories of Illness” variables did not allow to execute correlation analysis between these two variables and cognitive tests’ outcome scores in LHD group. Figure 1 in Supplement 2 presents the visualization of the significant relationships reported in this table between story types and cognitive tests’ scores in RHD group.

**Table 4.b.** Relationship between the thematic content and the cognitive functions (raw test outcome scores) in RHD.

|  |  | **Medical, Rho (*p*)** | **Physical, Rho (*p*)** | **Other, Rho (*p*)** | **Emotional, Rho (*p*)** | **Cognitive, Rho (*p*)** | **Circumstances of illness onset, Rho (*p*)** | **Strategies of coping with the illness, Rho (*p*)** | **Interpersonal, Rho (*p*)** | **Subjective theories of illness, Rho (*p*)** |
| --- | --- | --- | --- | --- | --- | --- | --- | --- | --- | --- |
| **Abstract Thinking** | WAIS-R(PL)  Similarities | -0.007 (0.979) | -0.080 (0.776) | -0.432 (0.161) | -0.350 (0.395) | 0.000 (1.000) | -0.550 (0.079) | -0.438 (0.205) | -0.113 (0.726) | -0.196 (0.641) |
|  | RHLB-PL  Inference Test | 0.240 (0.388) | 0.103 (0.715) | 0.144 (0.656) | -0.209 (0.619) | -0.216 (0.607) | 0.514 (0.106) | 0.167 (0.644) | -0.252 (0.430) | 0.510 (0.196) |
|  | Picture Metaphors Test | -0.344 (0.209) | -0.490 (0.070) | -0.357 (0.255) | -0.063 (0.882) | 0.587 (0.126) | -0.030 (0.930) | -0.216 (0.550) | -0.544 (0.067) | 0.616 (0.104) |
| **Attention and Psycho-**  **motor Speed** | TMT-A | -0.152 (0.589) | -0.145 (0.607) | 0.238 (0.456) | **0.662 (0.052)** | -0.234 (0.544) | 0.254 (0.451) | -0.060 (0.878) | 0.236 (0.461) | 0.290 (0.487) |
| **Executive Functions** | WCST  Correct Answers | 0.841 (0.036) | 0.522 (0.288) | -0.103 (0.870) | **-0.949 (0.051)** | **-0.939 (0.005)** | **-0.857 (0.029)** | -0.026 (0.966) | 0.154 (0.805) | -0.211 (0.789) |
|  | % Errors | -0.118 (0.662) | -0.199 (0.461) | 0.233 (0.443) | 0.532 (0.141) | 0.431 (0.246) | 0.201 (0.554) | 0.341 (0.334) | 0.208 (0.494) | -0.039 (0.921) |
|  | % Perseveration Errors | -0.600 (0.208) | -0.500 (0.312) | -0.100 (0.873) | 0.800 (0.200) | 0.617 (0.192) | **0.845 (0.034)** | -0.154 (0.805) | 0.000 (1.000) | 0.211 (0.789) |
|  | % Conceptual Answers | 0.771 (0.072 | 0.412 (0.417) | -0.300 (0.624) | 0.000 (1.000) | **-0.926 (0.008)** | **-0.845 (0.034)** | -0.205 (0.741) | 0.000 (1.000) | -0.211 (0.789) |
| **Language Functions** | WAIS-R(PL)  Comprehension | -0.113 (0.688) | -0.117 (0.677) | -0.507 (0.092) | -0.522 (0.185) | -0.18 (0.666) | -0.080 (0.815) | -0.476 (0.164) | **-0.642 (0.024)** | 0.111 (0.794) |
|  | RHLB-PL  Lexical-Semantic Test | 0.186 (0.507) | 0.131 (0.643) | -0.394 (0.205) | -0.531 (0.176) | 0.386 (0.345) | -0.370 (0.262) | -0.412 (0.236) | -0.448 (0.145) | 0.299 (0.472) |
|  | Emotional Prosody | 0.264 (0.362) | 0.224 (0.441) | -0.041 (0.899) | -0.683 (0.062) | -0.092 (0.829) | -0.433 (0.183) | 0.344 (0.331) | 0.292 (0.356) | -0.419 (0.302) |
| **Memory and Learning** | WAIS-R(PL)  Digit Span | -0.006 (0.982) | -0.168 (0.549) | -0.193 (0.548) | 0.325 (0.432) | 0.508 (0.199) | -0.310 (0.353) | 0.019 (0.959) | 0.128 (0.692) | 0.291 (0.485) |
|  | CVLT  A1-5 | -0.114 (0.685) | -0.159 (0.572) | 0.014 (0.965) | -0.596 (0.090) | -0.106 (0.785) | 0.125 (0.715) | 0.145 (0.710) | 0.118 (0.715) | -0.667 (0.071) |
|  | Free Recall Short Delay | -0.150 (0.595) | -0.402 (0.138) | -0.480 (0.114) | -0.430 (0.247) | 0.464 (0.208) | 0.157 (0.644) | -0.442 (0.234) | -0.516 (0.086) | -0.038 (0.929) |
|  | Free Recall Long Delay | 0.197 (0.482) | -0.336 (0.222) | -0.025 (0.938) | -0.207 (0.593) | 0.019 (0.961) | **0.615 (0.044)** | 0.061 (0.876) | -0.265 (0.404) | -0.168 (0.691) |
|  | Recognition | 0.054 (0.848) | -0.312 (0.258) | -0.420 (0.174) | -0.224 (0.562) | **0.650 (0.058)** | 0.106 (0.756) | -0.320 (0.401) | **-0.732 (0.007)** | 0.101 (0.812) |
| **Visuo-spatial Functions** | WAIS-R(PL)  Visual Puzzles | 0.512 (0.089) | 0.324 (0.304) | -0.339 (0.339) | -0.308 (0.553) | -0.359 (0.485) | -0.556 (0.120) | -0.248 (0.520) | -0.106 (0.770) | -0.241 (0.602) |

*Note.* Values presented in the table are Rho and uncorrected p-values for multiple comparisons. After applying Bonferroni adjustment for multiple comparisons, there were no significant correlations at the Bonferroni-adjusted *p*-value of <0.05. Figure 1 in Supplement 2 presents the visualization of the significant relationships reported in this table between story types and cognitive tests’ scores in RHD group.
