# Supplementary material for: In Search for the Meaning of Illness: Content of Narrative Discourse Is Related to Cognitive Deficits in Stroke Patients
Source: Front Psychol. 2021 Jan 18;11:548802. doi: 10.3389/fpsyg.2020.548802 (PMC7847845; doi:10.3389/fpsyg.2020.548802)
Supplement: Supplementary file 6 [file Table_5.DOCX]

**Table 5.a.** Relationship between the story type and the cognitive functions (raw test outcome scores) in LHD.

|  |  | **Quest story type (%), Rho (*p*)** | **Restitution story type (%), Rho (*p*)** | **Chaos story type (%), Rho (*p*)** |
| --- | --- | --- | --- | --- |
|  |  |  |  |  |
| **Abstract Thinking** | WAIS-R(PL)  Similarities | 0.146 (0.729) | -0.466 (0.244) | - |
|  | RHLB-PL  Inference Test | -0.108 (0.798) | 0.279 (0.504) | - |
|  | Picture Metaphors Test | -0.282 (0.498) | 0.185 (0.661) | - |
| **Attention and Psycho-**  **motor Speed** | TMT-A | -0.321 (0.482) | 0.378 (0.403) | - |
| **Executive Functions** | WCST  Correct Answers | 0.107 (0.819) | 0.107 (0.819) | - |
|  | % Errors | -0.107 (0.819) | 0.607 (0.148) | - |
|  | % Perseveration Errors | -0.214 (0.645) | -0.036 (0.939) | - |
|  | % Conceptual Answers | 0.107 (0.819) | -0.607 (0.148) | - |
| **Language Functions** | WAIS-R(PL)  Comprehension | 0.072 (0.866) | -0.386 (0.346) | - |
|  | RHLB-PL  Lexical-Semantic Test | 0.047 (0.912) | -0.298 (0.474) | - |
|  | Emotional Prosody | 0.417 (0.304) | 0.117 (0.782) | - |
| **Memory and Learning** | WAIS-R(PL)  Digit Span | 0.084 (0.844) | 0.373 (0.362) | - |
|  | CVLT  A1-5 | -0.563 (0.146) | 0.223 (0.596) | - |
|  | Free Recall Short Delay | -0.108 (0.798) | 0.285 (0.494) | - |
|  | Free Recall Long Delay | -0.342 (0.408) | 0.663 (0.073) | - |
|  | Recognition | -0.321 (0.438) | 0.075 (0.861) | - |
| **Visuo-spatial Functions** | WAIS-R(PL)  Visual Puzzles | -0.089 (0.840) | 0.185 (0.661) | - |

*Note.* Values presented in the table are Rho and uncorrected p-values for multiple comparisons. After applying Bonferroni adjustment for multiple comparisons, there were no significant correlations at the Bonferroni-adjusted *p*-value of <0.05. Low variability of data for “Chaos story type” variable did not allow to execute correlation analysis between this variable and cognitive tests’ outcome scores in LHD group.

**Table 5.b.** Relationship between the story type and the cognitive functions (raw test outcome scores) in RHD.

|  |  | **Quest story type (%), Rho (*p*)** | **Restitution story type (%), Rho (*p*)** | **Chaos story type (%), Rho (*p*)** |
| --- | --- | --- | --- | --- |
| **Abstract Thinking** | WAIS-R(PL)  Similarities | 0.247 (0.374) | -0.073 (0.797) | -0.244 (0.380) |
|  | RHLB-PL  Inference Test | 0.184 (0.512) | -0.285 (0.303) | 0.039 (0.889) |
|  | Picture Metaphors Test | -0.353 (0.197) | -0.072 (0.799) | 0.361 (0.186) |
| **Attention and Psycho-**  **motor Speed** | TMT-A | -0.113 (0.688) | 0.030 (0.914) | 0.157 (0.576) |
| **Executive Functions** | WCST  Correct Answers | 0.551 (0.257) | 0.725 (0.103) | **-0.893 (0.016)** |
|  | % Errors | 0.289 (0.278) | -0.275 (0.302) | 0.275 (0.303) |
|  | % Perseveration Errors | -0.714 (0.111) | -0.143 (0.787) | 0.516 (0.295) |
|  | % Conceptual Answers | 0.429 (0.397) | 0.771 (0.072) | **-0.880 (0.021)** |
| **Language Functions** | WAIS-R(PL)  Comprehension | 0.084 (0.765) | -0.084 (0.765) | -0.207 (0.460) |
|  | RHLB-PL  Lexical-Semantic Test | -0.305 (0.268) | 0.154 (0.585) | 0.143 (0.610) |
|  | Emotional Prosody | 0.133 (0.650) | 0.053 (0.858) | -0.063 (0.830) |
| **Memory and Learning** | WAIS-R(PL)  Digit Span | 0.128 (0.649) | -0.172 (0.541) | 0.160 (0.569) |
|  | CVLT  A1-5 | 0.181 (0.520) | -0.102 (0.718) | 0.106 (0.708) |
|  | Free Recall Short Delay | 0.020 (0.944) | -0.087 (0.758) | 0.063 (0.824) |
|  | Free Recall Long Delay | 0.049 (0.862) | -0.040 (0.888) | -0.044 (0.875) |
|  | Recognition | -0.067 (0.813) | -0.078 (0.784) | 0.130 (0.643) |
| **Visuo-spatial Functions** | WAIS-R(PL)  Visual Puzzles | 0.159 (0.622) | 0.152 (0.637) | -0.435 (0.157) |

*Note.* Values presented in the table are Rho and uncorrected p-values for multiple comparisons. After applying Bonferroni adjustment for multiple comparisons, there were no significant correlations at the Bonferroni-adjusted *p*-value of <0.05. Figure 1 in Supplement 2 presents the visualization of the significant relationships between story types and cognitive tests’ scores in RHD group.
