# Supplementary material for: In Search for the Meaning of Illness: Content of Narrative Discourse Is Related to Cognitive Deficits in Stroke Patients
Source: Front Psychol. 2021 Jan 18;11:548802. doi: 10.3389/fpsyg.2020.548802 (PMC7847845; doi:10.3389/fpsyg.2020.548802)
Supplement: Supplementary file 7 [file Data_Sheet_1.DOCX]

Appendix 1

Example utterances of the identified main topics.

Medical:

- 'At the hospital they examined me fast, they measured my blood pressure, did electrocardiography, and diagnosed that I have the... some kind of the... beginning of stroke. So that they took me to another hospital. There again they examined me, did tomography and all those tests.' (HD, female, years old)

Physical:

- 'Paresis of left upper and lower extremities, there are no other symptoms. That is, I don't walk... properly yet, I don't move my left arm properly, that's all, other than that nothing happens.' (RHD, female, 59 years old)

Other:

- 'He [priest] was frustrating me, especially when it comes to the subject of church organisation. Our church, you know, it is from the times when they used to build from.. this material... from a very heavy wood. It is a very antique church, but recently he [priest] was renovating it and the altars, and I don't know if he did it maliciously, or just he lack sense of good taste..' (RHD, female, 83 years old)

Interpersonal:

- 'Now they [children] see that they have a father more than before. One time one daughter comes, another time my son-in-law comes and takes me home with him. So they all want to help me.' (RHD, male, 53 years old)

Emotional:

- 'I will try to be less nervous... so that it would come back when I was less nervous, because now I am very nervous, compulsive, but also not to all people.' (RHD, female, 70 years old)

Circumstances of illness onset:

- 'I came back from the shopping as usual. I lied down on the couch, took about two-hours nap. I got up from the couch, I wanted to go out on the balcony, but my leg already got stiff, and my daughter, I called for my daughter, daughter called the ambulance.' (RHD, male, 53 years old)

Subjective theories of illness:

- 'They [neighbours], I believe, have caused my current illness, because those people have made me terribly nervous.' (RHD, female, 83 years old)

Cognitive:

- 'You know, I see problems in speaking, and in in walking, in concentration, what else, in concentration, in looking from thoughts in myself. But as I say it depends on the day, there are better days that I have better concentration, but there are days that I have terrible concentration.' (LHD, male, 45 years old)

Strategies of coping with the illness:

- 'I came back home [from the hospital], and so what to do next? I must improve my speech and all of that, so I said that at home I won't achieve anything, I won't learn anything. But so it happened that my friend told me that maybe I could come to this rehabilitation centre. So I sent my documents and very fast, within three days, got accepted.' (LHD, female, 67 years old)

Appendix 2

Example utterances classified due to Frank's (1995) typology.

Restitution story type:

- 'They [doctors] had this method, thrombolysis, and they were treating me with this method. […] For some time I used to be in a wheelchair, now I can walk and I am very grateful to my doctors for it. I have left arm, which is paralysed, to treat yet. But I hope it's going to get better just like the leg.' (RHD, female, 53 years old)
- '[…] I had here [at the hospital] exercises for arm, I learned to write, other than that I had here gymnastics on the machines, bicycle, I exercised my legs on a special machine. Specially those exercises I had every day, every day systematically, and I came to such a mastery, one could almost say that I came back to health.' (LHD, female, 67 years old)

Quest story type:

- 'I don't have to do the choirs, my wife does everything [laughing], that's one plus. Second plus: before the stroke I used to stutter, now I don't. These are two basic pluses. There are no minuses, one hand is enough to operate and everything is fine.' (LHD, male, 53 years old)
- 'Our Lady gave me a warning, that I have to care for myself, because I have always considered myself a “hero”. I used to say “I'm made from iron, nothing can touch me...”, but it [stroke] did.' (RHD, female, 70 years old)

Chaos story type:

- 'Now I learned what a stroke is […] Now I see that it is a horrifying illness, [silence] very horrifying. I have the left side paralysed, I lost [silence]' (RHD, male, 59 years old)
- 'And this Anna little also butterflies are flying so I would go with her and explain and flowers are blooming, this is what I lack, so that I can walk and explain her what it is [silence] And mushroom season is coming, I like to pick mushrooms, but I don't know if I will walk, and I see it all, and I cannot manage it, this is the worst for me. […] This illness, that there is no cure for it. A flu, a person will get better, will walk. But this you cannot unfortunately, there is no medicine for it.' (RHD, female, 57 years old)
